# Supplementary figures and images for: Cleaner wrasse Labroides dimidiatus perform above chance in a “matching-to-sample” experiment
Source: PLoS One. 2022 Jan 31;17(1):e0262351. doi: 10.1371/journal.pone.0262351 (PMC8803161; doi:10.1371/journal.pone.0262351)

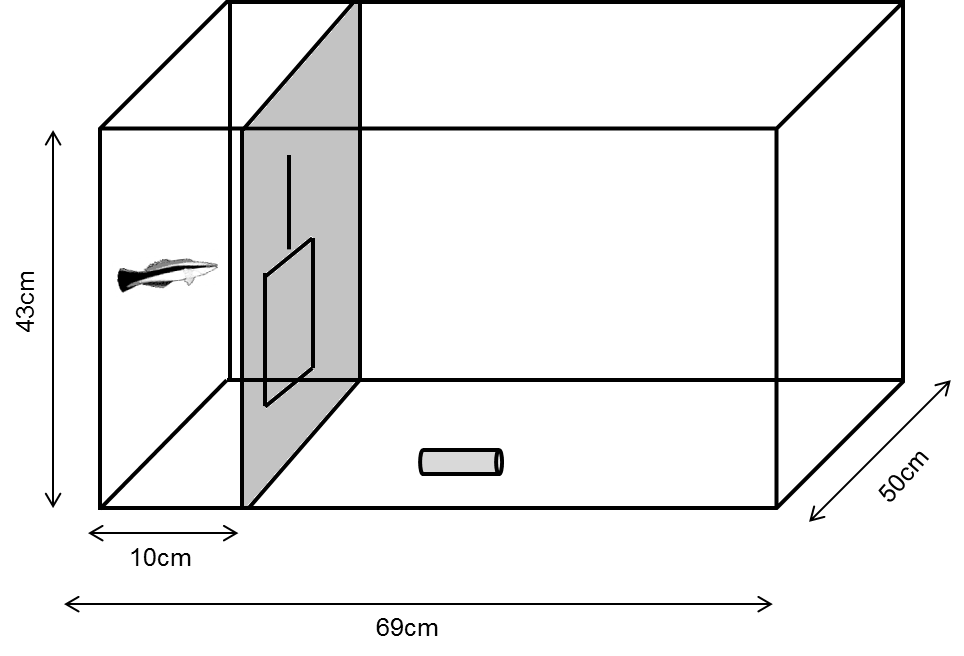

Supplement: S1 Fig — The “resting” area is on the left where the fish is waiting behind a see-through grid barrier with a door in the middle. On the right is the “experimental” compartment where the experimental plate is displayed during the trial. (TIF) [file pone.0262351.s001.tif]

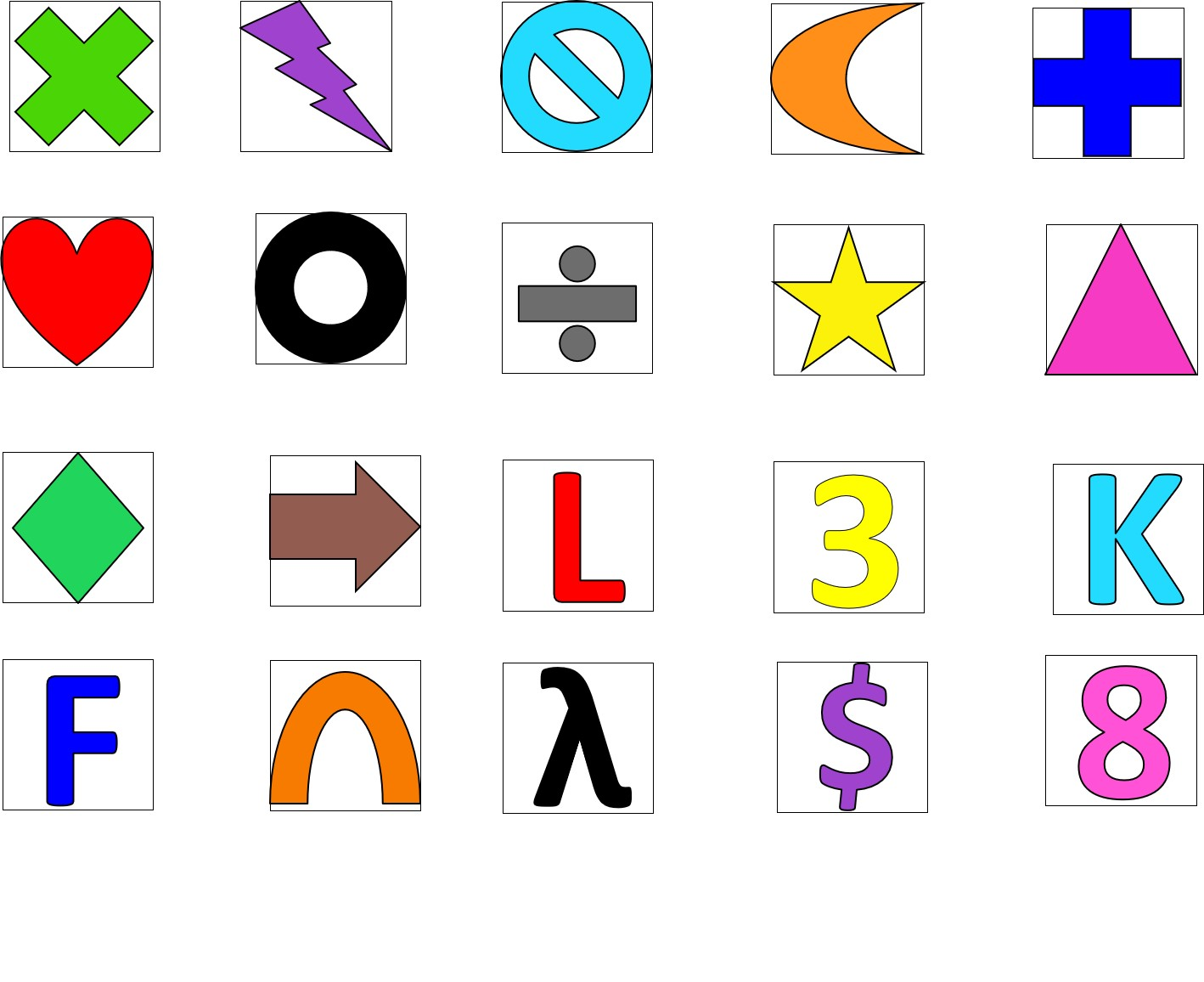

Supplement: S2 Fig — The symbols were drawn on a 3 x 3cm white paper sheet. (TIF) [file pone.0262351.s002.tif]
