# Supplementary material for: Cleaner wrasse Labroides dimidiatus perform above chance in a “matching-to-sample” experiment
Source: PLoS One. 2022 Jan 31;17(1):e0262351. doi: 10.1371/journal.pone.0262351 (PMC8803161; doi:10.1371/journal.pone.0262351)
Supplement: S1 Table — (PDF) [file pone.0262351.s003.pdf]

Reference	Species (n)	Task	Stimuli	Pre-training and/or training	N° training trials	Transfer test	N° transfer trials	Success/fail
**Finch, 1942**<br><br>**(by Oden et al., 1988)**	Chimpanzee (4)	MTS	?	two training stimuli	600 – 1,000	new stimuli	similar to Oden et al, 1988: 24 trials	success
**Nissen et al., 1948**<br><br>**(by Oden et al., 1988)**	Chimpanzee (4)	MTS	?	two training stimuli	600 – 1,000	new stimuli	similar to Oden et al, 1988: 24 trials	success
**Blough, 1959**	Pigeons (4)	MTS	light	two training stimuli<br><br>steady light / flicker light	500 – 1,000	new stimuli<br><br>(for 20 presentations)	8000<br><br>90% correct	2/4 succeeded
**Goldman and Shapiro, 1979**	Goldfish (5+5)	simultaneous MTS<br><br>(n = 1)	light	Pre-training: white light in the middle, white light both side, touch and receive food	300 – 500	same stimuli	8400<br><br>60%	success
				Training: 3 stimuli: red, green and blue light	8,400<br><br>60%			
**Zerbolio et Royalty, 1983**	Goldfish (24+24)	MTS<br><br>(n = 4)	light	12 with red/green<br><br>12 with blue/yellow<br><br>30 days<br><br>40trials/day<br><br>5days/week	840 on 1,200 trials total	2x 8 (n = 2) new stimuli<br><br>2x 8 (n = 2) same stimuli<br><br>30 days<br><br>40trials/day<br><br>5days/week	600 on average	success

**D'Amato et al, 1985**	Capuchin monkeys (8)	MTS	symbol	pre-training: sample - 1 stimulus showed		3 visual stimuli: coloured disk red, yellow and blue	240 - 624	success
				MTS training: simultaneous MTS procedure, only 2 stimuli used (shape)<br><br>criterion: 2x 90% on 2 successive blocks of 48 trials	624 – 4,673			
**Wright et al, 1988**	Pigeons (4)	MTS	symbol	pre-training: 2 stimuli (arrow + apple)				
				training: 35 trials (1st session), grain placed on the correct comparison stimulus before the choice				
			Trial unique	152 different stimuli presented once daily<br><br>76 trials per day --> 75% over 360 training sessions (18 months; 360 presentations)	27,360	transfer: 10 trials/session 2 novel stimuli used at each trial<br><br>25 warm up trials // 10 transfer trials incorporating in remaining 51 training trials + 40 stimuli (2 consecutive transfer sessions)<br><br>second transfer test with 40 test stimuli	7000<br><br>60%	success<br><br>(n = 2)
			2 stimuli	train with 2 stimuli	1,216	Same as above	7000	fail

				75% 16 sessions (1216 presentations)<br><br>76 trials<br><br>criterion: reach 75% or more --> tested to transfer				(n = 2)
**Oden et al., 1988**	Infant chimpanzees (4)	MTS	objects	Pre-trained to handle 2 objects (cup and lock)<br><br>when putting an object into a baking tin rewarded via hugs and congratulations	642 – 1,000	2 new stimuli<br><br>session of 12 trials<br><br>reward with food and congratulations even when incorrect choice to keep them motivated<br><br>6 transfer tests so 12 novel objects in total	24<br><br>mean % correct 67.7 - 85.4 %	success
				training: give one object have to put it in a baking tin, then two alternative objects were placed 20cm apart and equidistant from the pan<br><br>session of 12-24 trials / ITI = mean of 30s / 1-3 times daily 5 times a week<br><br>criterion level: at least 10/12	816<br><br>(mean)			
**Herman et al., 1989**	Bottlenose dolphin (1)	MTS	objects	4-5 stimuli<br><br>16-20 trials per session<br><br>2 sessions per day<br><br>simultaneous MTS as training: 72 trials over 6 days	432<br><br>81%	Same stimuli<br><br>2-4 sessions for a total of 36-63 trials overall 83.3% correct<br><br>NO transfer trials	564<br><br>83.3%	success

**Pack et al, 1991**	Sealion (1)	generalized MTS	symbol	MTS training with 2 stimuli<br>24 trials per session<br>criterion 2x 90% in a row	269	Same stimuli<br>transfer test: add 1 novel stimulus compared to the 2 previous one from the training phase<br>criterion 2x 90% in a row	72	success
				with 3 stimuli	503			
**Iversen, 1993**	Rats (3)	MTS	light	pre-training: steady / blinking<br>sample in the middle, rat press it, stimulus (same) on one of the side, press, food<br>2x 100 trials	200	Same stimuli<br>80% after 25 sessions<br>90% after 50 sessions for Rat 1, 60 for Rat 2 and 4 for Rat 3<br>NO transfer trials	1000 – 1500<br>60%	success
				MTS training: simultaneous MTS / same stimuli as in pre-training<br>1 session = 100 trials + number of repeated trial	5,000			
**Giurfa et al, 2001**	Honeybees (360)	MTS	Colour<br>grating	2 stimuli (colour)<br>Y-maze, entrance encountered sample stimulus<br>then stimuli A and B have to choose in decision chamber	60	2 new stimuli (grating)	60	success
**Bodily et al., 2008**	Pigeons (5)	simultaneous MTS	cartoon images<br>computer	3 pre-training stimuli<br>84 trials/session	2,352	transfer trial with novel stimuli each time<br>4 consecutive transfer sessions conducted	384	success

				3 same stimuli as pre-training<br><br>training: 96 trials/day<br><br>12 combinations configuration<br><br>>85% correct on 2 consecutive sessions with CP --> without CP: >85% correct in 1 session	672	48 novel pairs		
**Truppa et al., 2010**	Capuchin monkeys (6)	Simultaneous MTS	symbol	2 stimuli used<br><br>12 consecutive correct trials no more than 3 corrections	10,852	10 transfer tests composed of 4 sessions	1812 - 48 (decreasing over test)	success
						final transfer test<br><br>single 100 trials session with new stimuli	59%	
**Gierszewski et al, 2013**	Cichlids (9)	Simultaneous MTS	symbol	2 stimuli presented<br><br>10 trials per session, 2 per day<br><br>criterion 3x 70% in a row<br><br>35-40 sessions	350 - 400	using cross and circle	within 400 trials	fail
**Newport et al, 2014**	Archerfish (7)	Simultaneous MTS	symbol	As Goldman and Shapiro, 1979<br><br>Pre-training: white light in the middle, white light both side, touch and receive food<br><br>Training: 3 stimuli: red, green and blue light		3 stimuli	800	fail

**Bloch et al, 2019**	Zebrafish (10-12 per experiment)	Simultaneous MTS	Colour	Colour discrimination as training<br><br>10 trials per session, 1 per day<br><br>ITI = 10s<br><br>criterion: 3x 70% or above in a row	70 – 80	Simultaneous MTS as training<br><br>Same colour stimuli<br><br>criterion: 3x 70% or above in a row	120 – 150	success
