# Supplementary material for: Cleaner wrasse Labroides dimidiatus perform above chance in a “matching-to-sample” experiment
Source: PLoS One. 2022 Jan 31;17(1):e0262351. doi: 10.1371/journal.pone.0262351 (PMC8803161; doi:10.1371/journal.pone.0262351)
Supplement: Attachment — Submitted filename: Response to Reviewers.docx [file pone.0262351.s004.docx]

CC: adrian.dyer@rmit.edu.au

PONE-D-21-17431
Cleaner wrasse Labroides dimidiatus perform above chance in a "Matching-to-sample" experiment
PLOS ONE

Dear Dr. Aellen,

Thank you for submitting your manuscript to PLOS ONE. After careful consideration, we feel that it has merit but does not fully meet PLOS ONE’s publication criteria as it currently stands. Therefore, we invite you to submit a revised version of the manuscript that addresses the points raised during the review process.

So far I have had one independent expert on animal behaviour review the manuscript, and I have also carefully read the work. I agree with R1 that the work is potentially of interest to publish, but clarity of explanations and writing can be improved a lot (see reveiwer report). In particular, I agree with R1 that readers need a more complete explanation of MTS (matching to sample)for which you use simultaneous presentation, and how delayed MTS (ie DMTS) experiments has been more frequently used in other animal species, at least in recent times. DMTS requires buth longer term memory formation of rule, and working memory (WM) of samples in a particular instance. The simultaneous MTS thus is not directlly comparable to DMTS; at least you need to clearly explain differences for readers and how such differences influence your experimental interpretation(s).

I also found the line "Fish were fed ad libitum for five minutes 20 minutes prior to the start of the experiment." confusing as to what this meant.........please use a lot more care so a general readership can fully understand your paper. I also was confused by s3 Figure which I cant see mentioned in main manuscript. Can you please take much more care in paper presentation and message? I have allowed time for a major revision to enable this. If you can do a good job I will then ask R1 and another expert to review the revised paper.

Dear Dr Dyer, we would like to thank you for your comments. We considered each input and hope that the new version will be more suitable and reach your journal standards in PLOS ONE.

Please submit your revised manuscript by Aug 28 2021 11:59PM. If you will need more time than this to complete your revisions, please reply to this message or contact the journal office at plosone@plos.org. When you're ready to submit your revision, log on to https://www.editorialmanager.com/pone/ and select the 'Submissions Needing Revision' folder to locate your manuscript file.

Please include the following items when submitting your revised manuscript:

- A rebuttal letter that responds to each point raised by the academic editor and reviewer(s). You should upload this letter as a separate file labeled 'Response to Reviewers'.
- A marked-up copy of your manuscript that highlights changes made to the original version. You should upload this as a separate file labeled 'Revised Manuscript with Track Changes'.
- An unmarked version of your revised paper without tracked changes. You should upload this as a separate file labeled 'Manuscript'.

If you would like to make changes to your financial disclosure, please include your updated statement in your cover letter. Guidelines for resubmitting your figure files are available below the reviewer comments at the end of this letter.

If applicable, we recommend that you deposit your laboratory protocols in protocols.io to enhance the reproducibility of your results. Protocols.io assigns your protocol its own identifier (DOI) so that it can be cited independently in the future. For instructions see: [http://journals.plos.org/plosone/s/submission-guidelines#loc-laboratory-protocols](http://journals.plos.org/plosone/s/submission-guidelines#loc-laboratory-protocols). Additionally, PLOS ONE offers an option for publishing peer-reviewed Lab Protocol articles, which describe protocols hosted on protocols.io. Read more information on sharing protocols at [https://plos.org/protocols?utm_medium=editorial-email&utm_source=authorletters&utm_campaign=protocols](https://plos.org/protocols?utm_medium=editorial-email&utm_source=authorletters&utm_campaign=protocols).

We look forward to receiving your revised manuscript.

Kind regards,

Adrian G Dyer, Ph.D.
Academic Editor
PLOS ONE

Journal Requirements:

When submitting your revision, we need you to address these additional requirements.

1. Please ensure that your manuscript meets PLOS ONE's style requirements, including those for file naming. The PLOS ONE style templates can be found at 
https://journals.plos.org/plosone/s/file?id=wjVg/PLOSOne_formatting_sample_main_body.pdf and 
https://journals.plos.org/plosone/s/file?id=ba62/PLOSOne_formatting_sample_title_authors_affiliations.pdf

Thank you, we will carefully follow the journal guidelines.

2. Thank you for stating the following financial disclosure: 
 [The study was supported by the Swiss National Science Foundation (grant numbers 31003A_153067 and 310030B_173334/1) to R.B. 
NO]. 
a) Please provide an amended Funding Statement that declares *all* the funding or sources of support received during this specific study (whether external or internal to your organization) as detailed online in our guide for authors at <http://journals.plos.org/plosone/s/submit-now>.

We took good notes of it and will do the necessary, thank you.

b) Please state what role the funders took in the study.  If any authors received a salary from any of your funders, please state which authors and which funder. If the funders had no role, please state: "The funders had no role in study design, data collection and analysis, decision to publish, or preparation of the manuscript."

Please send your amended statements by return email; we will change the online submission form on your behalf.

The amended statements have been returned by email, thank you.

3. Thank you for stating the following in the Acknowledgments Section of your manuscript: 
[We are grateful to the Gump research station in Moorea (French Polynesia) for letting us work in their station. We are particularly thankful to the directors and the staff for their unconditional help and great friendship. We thank Léonore Bonin for fish capturing. We acknowledge Dr. Christèle Borgeaud for English corrections and valuable comments. We thank Radu Alexandru Slobodeanu for statistical help and support. The study was supported by the Swiss National Science Foundation (grant numbers 31003A_153067 and 310030B_173334/1) to R.B.]
We note that you have provided funding information that is currently declared in your Funding Statement. However, funding information should not appear in the Acknowledgments section or other areas of your manuscript. We will only publish funding information present in the Funding Statement section of the online submission form. 
Please remove any funding-related text from the manuscript and let us know how you would like to update your Funding Statement.

Thank you for your comment. We did remove the funding-related text from the manuscript and updated the Acknowledgments section accordingly on L. 314 - 319.

4. Please include captions for your Supporting Information files at the end of your manuscript, and update any in-text citations to match accordingly. Please see our Supporting Information guidelines for more information: http://journals.plos.org/plosone/s/supporting-information.

We did add the captions for the Supporting Information at the end of the manuscript and updated them in the text accordingly, thank you.

[Note: HTML markup is below. Please do not edit.]

Preamble to our replies to the specific referee comments below: we would like to thank the referee for his/her highly useful comments. The different comments have led to substantial changes in the introduction as demanded, and we trust that our revised version make the paper clearer.

Reviewers' comments:

Reviewer's Responses to Questions

**Comments to the Author**

1. Is the manuscript technically sound, and do the data support the conclusions?

The manuscript must describe a technically sound piece of scientific research with data that supports the conclusions. Experiments must have been conducted rigorously, with appropriate controls, replication, and sample sizes. The conclusions must be drawn appropriately based on the data presented.

Reviewer #1: Yes

2. Has the statistical analysis been performed appropriately and rigorously?

Reviewer #1: I Don't Know

3. Have the authors made all data underlying the findings in their manuscript fully available?

The [PLOS Data policy](http://www.plosone.org/static/policies.action#sharing) requires authors to make all data underlying the findings described in their manuscript fully available without restriction, with rare exception (please refer to the Data Availability Statement in the manuscript PDF file). The data should be provided as part of the manuscript or its supporting information, or deposited to a public repository. For example, in addition to summary statistics, the data points behind means, medians and variance measures should be available. If there are restrictions on publicly sharing data—e.g. participant privacy or use of data from a third party—those must be specified.

Reviewer #1: No

Sorry we do not understand, under the ‘Statistical analyses’ part of the manuscript, it is stipulated that ‘All data and the R-scripts to reproduce the analyses are available at <https://figshare.com/s/4ed4d7c5bcc750500de9>.’ On L. 213-214.

4. Is the manuscript presented in an intelligible fashion and written in standard English?

PLOS ONE does not copyedit accepted manuscripts, so the language in submitted articles must be clear, correct, and unambiguous. Any typographical or grammatical errors should be corrected at revision, so please note any specific errors here.

Reviewer #1: No

5. Review Comments to the Author

Please use the space provided to explain your answers to the questions above. You may also include additional comments for the author, including concerns about dual publication, research ethics, or publication ethics. (Please upload your review as an attachment if it exceeds 20,000 characters)

Reviewer #1: In this study, the authors tested the ability of cleaner fish to succeed in a simultaneous matching-to-sample task. Such task is classically used to test for rule learning based on a ‘same’ abstract relational concept. This cognitive ability has been established in many animal species from mammals to insects. However, while some fish species appeared able to master this task (zebrafish and goldfish), others were not (e.g. archerfish). The current study thus proposes to further explore fish concept learning capacities by testing an additional species. This question is scientifically valid and of interest as I consider that the comparative cognition field certainly gains from more model species to be tested for key cognitive tasks. The results demonstrate great individual variability but with success of a few individuals. These results support the existence of an ability for solving matching-to-sample task in this species in particular as the procedure chosen is not classic and may have had detrimental effect on performance. Indeed, usually, the sample is not longer present when the subject has to make a choice between a stimulus identical to the sample and an alternative stimulus. In addition, only a limited number of samples are used in standard procedures at least during the acquisition phase while in the current study, each trial presented a novel stimulus. These key differences may have induced attentional bias and noise which prevented most subjects to extract the common rule between trials. Proposing alternative procedures is of high interest to better understand the underlying processes but I regret that the authors did not test in parallel the classical procedure for comparison. With the current data, it is indeed impossible to compare the cleaner fish performances with those of other species.

We would like to thank you for your comments. We agree that we cannot compare our study with others on the simultaneous matching-to-sample task as we did change the procedure substantially. However, we believe that this new procedure might be of great interest for further studies. Indeed, it is known that the use of novel stimuli over trials is the only way to determine whether animals achieved abstract concept learning. According to this view, using the same stimuli repeatedly over trials may only yield indicative results. While some studies have supplemented the initial solving with new stimuli afterwards, on an individual level it could still be a case of ‘learning to learn’. Furthermore, it has been shown that the fact to utilize an increased number of various stimuli enhance the gain of relational ability. We propose that our procedure avoids all these potential shortcomings; if subjects perform above chance it must be based on abstract concept learning. We do agree that to study cleaner fish in the ‘standard’ procedure of simultaneous MTS might be a nice future study. We say so explicitly in the new version in the discussion.
Other comments:
Introduction: The introduction needs quite a substantial editing. Some sentences are difficult to read, some others are incorrect or imprecise. I recommend the authors to invest more effort in drafting the introduction in a novel version of the manuscript.

Thank you for your input. We revised the introduction and hopefully made our writing clearer. Indeed, we changed the order of points and wrote more explicitly about various aspects.

A few examples:
l. 35 : do you mean a shaping procedure ? It is not clear what you mean by operant conditioning. Your description does not allow to understand what is the procedure to study the ability of animals to learn abstract concepts. Please revise the lines 34-37

Thank you for your comment. We are now much more explicit about the experimental paradigm, which should make things clear on L. 57 – 63.

l. 46 : I do not understand this sentence. ‘concepts are represented by the relation of abstract rules to novel stimuli’ ??

Thank you for your comment. We chance the sentence accordingly on L. 50 - 53.

l. 49-53 : Difficult to follow, please revise for improved clarity

We change this sentence accordingly to clarify the procedure on L. 78 – 80. Thank you

l.57 : The reference 27 seems fully out of context here. Could you please provide references of studies using simultaneous MTS to test for same/difference concept learning ?

We apologize, the right references were added on L. 75. Thank you

l. 58: Incorrect references as they used a DMTS procedure and not a simultaneous MTS
…

Thank you for your remark. We change the reference accordingly on L. 75.

Methods
l. 164 : Why did you feed the fish ab libitum before the experiment. This should induce a lack of motivation for the food reward.

Thank you for your comment. ‘We changed the word ‘ad libitum’ as we simply offered food for 5 min. Cleaner fish eat 11 hours during the day and hence have an adapted digestion. This implies that they are hungry in the morning, after 13h without food. The goal of giving them some food prior to the start of the trials was to avoid that they are so hungry that they do not pay attention to the stimuli. The 10 trials per day were spread over 5-6 h, so any lack of motivation due to the feeding would have affected at best the first trial (L. 191 – 197).

l. 165 : Why did you let the food plate in place for 2 hours ?
I suggest to provide a video as supporting information to help the reader to better picture the whole procedure.

Thank you for your comment. We left the plate for about 2 hours after the trials to make sure that fish did eat enough for the day (L. 191 - 197). Food intake during trials was quite low.

Discussion
l.228 : There is at least one study, the one in pigeons which is described a few sentences later (ref 12)

Thank you for your remark. In the study with the pigeons, they did use 76 stimuli but those stimuli were used every day. Here in our study, no same stimuli were shown over the 200 trials (so 400 stimuli were used in total without being used twice) (L. 269 – 271).

Bibliography : please check the references formatting as not consistent between references and does not follow the journal guidelines.

Thank you for your comment. The bibliography was carefully checked and the formatting respected to follow the journal guidelines (Vancouver style) on L. 323 and so on.

6. PLOS authors have the option to publish the peer review history of their article ([what does this mean?](https://journals.plos.org/plosone/s/editorial-and-peer-review-process#loc-peer-review-history)). If published, this will include your full peer review and any attached files.


If you choose “no”, your identity will remain anonymous but your review may still be made public.


**Do you want your identity to be public for this peer review?** For information about this choice, including consent withdrawal, please see our [Privacy Policy](https://www.plos.org/privacy-policy).

Reviewer #1: No

[NOTE: If reviewer comments were submitted as an attachment file, they will be attached to this email and accessible via the submission site. Please log into your account, locate the manuscript record, and check for the action link "View Attachments". If this link does not appear, there are no attachment files.]

While revising your submission, please upload your figure files to the Preflight Analysis and Conversion Engine (PACE) digital diagnostic tool, [https://pacev2.apexcovantage.com/](https://pacev2.apexcovantage.com/). PACE helps ensure that figures meet PLOS requirements. To use PACE, you must first register as a user. Registration is free. Then, login and navigate to the UPLOAD tab, where you will find detailed instructions on how to use the tool. If you encounter any issues or have any questions when using PACE, please email PLOS at [figures@plos.org](mailto:figures@plos.org). Please note that Supporting Information files do not need this step.
